# Overexpression of hypoxia-inducible factor 1α and excessive vascularization in the peri-implantation endometrium of infertile women with chronic endometritis

**DOI:** 10.3389/fendo.2022.1001437

**Published:** 2022-12-01

**Authors:** Zhenteng Liu, Xuemei Liu, Fenghua Li, Yuxia Sun, Lili Yu, Wei Zhang, Ping Zhu, Ding Ma, Xinrong Wang, Shoucui Lai, Hongchu Bao

**Affiliations:** Department of Reproductive Medicine, Yantai Yuhuangding Hospital Affiliated to Qingdao University, Yantai, Shandong, China

**Keywords:** chronic endometritis, *in vitro* fertilization, hypoxia-inducible factor 1α, VEGFA, infertility, endometrial receptivity, endometrial vascularization

## Abstract

**Objective:**

Chronic endometritis (CE) contributes to impaired endometrial receptivity and is closely associated with poor *in vitro* fertilization (IVF) outcomes. However, the mechanisms underlying CE are unclear. Here, we investigated the role of the hypoxic microenvironment and endometrial vascularization in the peri-implantation endometrium of infertile women with CE.

**Methods:**

This retrospective study involved 15 fertile women and 77 infertile patients diagnosed with CE based on CD138+ ≥1/10 high-power fields (HPFs). The CE patients were divided into Group 1 (CD138+ 1–4/10 HPFs, 53 cases) and Group 2 (CD138+ ≥5/10 HPFs, 24 cases). The expression levels of hypoxia-inducible factor 1α (HIF1α), vascular endothelial growth factor A (VEGFA), and vascular endothelial growth factor receptor 2 (VEGFR2) in peri-implantation endometrium were assessed by qRT-PCR and western blot analyses. Spatial levels of HIF1α, VEGFA, and VEGFR2 in various endometrial compartments was determined using immunohistochemistry and *H*-score analysis. Microvascular density (MVD) was determined using CD34 staining and scored using Image J. Finally, we used qRT-PCR to assess changes in the expression of HIF1α, VEGFA, and VEGFR2 in CE patients after treatment with first-line antibiotics.

**Result(s):**

Relative to Group 1 and control group, during the implantation window, protein and mRNA levels of HIF1α, VEGFA, and VEGFR2 were markedly high in Group 2 (*P*<0.05). *H*-score analysis showed that HIF1α, VEGFA, and VEGFR2 in the luminal, glandular epithelium, and stromal compartments were markedly elevated in Group 2, comparing to control group and Group 1 (*P*<0.05). Moreover, markedly elevated MVD levels were observed in Group 2. Notably, the above indexes did not differ significantly in the control group versus Group 1. Treatment with antibiotics significantly suppressed the endometrial HIF1α and VEGFA levels in CE-cured patients.

**Conclusion(s):**

Here, we for the first time report the upregulation of HIF1α, VEGFA, and VEGFR2, as well as excessive endometrial vascularization in the peri-implantation endometrium of CE patients. Our findings offer new insights into reduced endometrial receptivity in CE-associated infertility.

## Introduction

Chronic endometritis (CE) refers to the continuous and subtle infections of the endometrial mucosa (1), which are mainly thought to be caused by various bacteria, such as *Escherichia coli, Staphylococcus* spp.*, Mycoplasma, Chlamydia, Streptococcus* spp.*, and Ureaplasma*. However, CE etiology remains unclear (2). Currently, the gold standard for CE diagnosis is fluid hysteroscopy combined with endometrial biopsy and histopathological identification based on immunostaining for syndecan-1 (CD138), a plasma cell marker (3–5). However, consensus on the cutoff values for identifying clinical CE is lacking.

Mounting evidence indicates that CE adversely affects reproductive health and pregnancy outcomes after *in vitro* fertilization and embryonic transfer (IVF-ET), such as repeated implantation failure (RIF) (6–8), recurrent miscarriage (RM) (9–11), and preterm labor (12). Several studies have reported that treatment with oral antibiotics significantly improves IVF outcomes in women with CE (10, 13–15). Recent findings indicate that CE influences the level and population of immunocompetent cells that infiltrate the endometrium (7, 16, 17). Additionally, CE dysregulates the expressions of various endometrial receptivity-associated genes in proliferative and mid-luteal phases (18, 19). CE also adversely affects endometrial receptivity by negatively impacting normal endometrial decidualization by modifying the expression of sex steroid receptors (20) and shifting cytokine milieu towards proinflammatory immune responses by impairing autophagy (21). Recent studies involving endometrial receptivity analysis (ERA) have reported that CE modifies the individual window of implantation (WOI), leading to embryo–endometrial asynchrony (22). But, the pathomechanisms by which CE exerts its negative effects on implantation and pregnancy, especially endometrial angiogenesis dysregulation, are unclear.

During the peri-implantation period (cycle days 20–24), harmonious endometrial angiogenesis is critical for the supply of oxygen and nutrients for cell growth and successful embryo implantation (23). During this time, the endometrial microenvironment has low oxygen levels and normal oxygen homeostasis is vital for early development of the trophoblast (24, 25). Hypoxia-inducible factor-1α (HIF1α) is key in adaptation of cell to hypoxia and is expressed exclusively during the menstrual and secretory phases (26). HIF1α mediates inflammatory response and vasculogenesis by activating the transcription of angiogenic genes (27). We have previously reported that overweight PCOS is associated with significantly reduced HIF1α expressions in mid−secretory phases of the human endometrium, which may be associated with reduced endometrial receptivity (28). Chen, X., et al. reported that HIF−1α levels in endometrial luminal epithelium and stroma in women with recurring miscarriage are high, relative to fertile controls. However, the role of HIF1α in CE pathogenesis is unknown.

Vascular endothelial growth factor A (VEGFA), a HIF1α target, is the main stimulator of angiogenesis and normal VEGFA levels are necessary for endometrial receptivity (29). To exert its effects, VEGFA binds the three tyrosine kinase receptors, VEGFR-1, -2, and -3 (29). VEGFR2, which exhibits the highest proangiogenic activity, is mainly expressed in luminal epithelial as well as in endometrial glandular cells, and in myometrial smooth muscle cells (30). Additionally, CD34 is a widely used immunohistochemical indicator of microvascular density (MVD: number of vessel profiles/mm^2^) in endometrial sections (31, 32). Endometrial vascularity has also been measured by quantifying the above indexes in multiple situations, such as recurrent miscarriage and recurrent implantation failure (23, 32, 33). Notably, the role of angiogenesis and vascular distribution at the time of embryo implantation in CE patients is unclear.

To improve our understanding of CE and uncover potential therapeutic strategies, a systemic investigation of endometrial oxygen homeostasis and vascular pathology is required. To the best of our knowledge, our study is the first to investigate the expression of HIF1α, VEGFA, and VEGFR2, as well as endometrial vascular features during the precisely timed implantation window in CE patients versus fertile women without CE.

## Materials and methods

### Subjects

Ethical approval for this retrospective case control study was granted by the ethical committee of Yantai Yuhuangding Hospital Affiliated to Qingdao University. Study participants were required to sign written informed consents after receiving details on the study during their first appointment. A total of 77 infertile women with CE diagnosed using hysteroscopy, histological identification of plasma cells and CD138 immunohistochemistry were involved into the study. These participants had been trying to conceive for ≥1 year and were referred to the reproduction medical center of Yantai Yuhuangding Hospital Affiliated to Qingdao University between July 2019 and February 2022 with unexplained infertility. During the same period, 15 healthy age-matched women who had conceived naturally, had ≥1 live births, and had no history of spontaneous early pregnancy loss, were recruited as the control group during clinic visits for physical examination or contraception counseling. Other inclusion criteria were, a) age of 22–38 years, b) regular menstrual cycles (26–33 days), c) no history of endometriosis or adenomyosis, d) no autoimmune or thrombotic disease, e) no previous tuberculosis diagnosis, and f) no immunotherapy, steroid hormone treatment, or antibiotic management during the preceding three months.

### Hysteroscopy and endometrial biopsy

**Figure 1:**
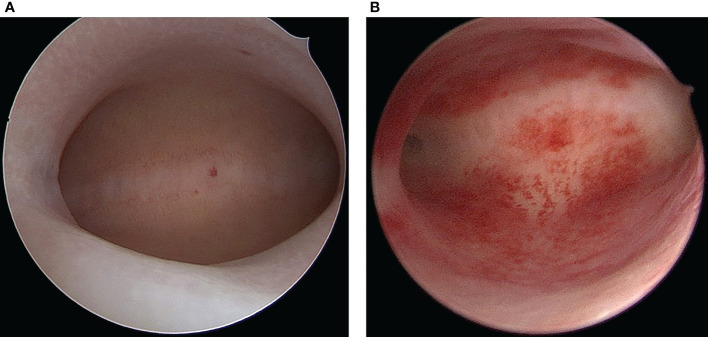
Hysteroscopic finding of normal endometrium **(A)** and chronic endometritis **(B)**.

To detect luteinization hormone (LH) surge, from day 9 of the menstrual cycle, all participants underwent a urine dipstick test daily. Hysteroscopy using a forward-oblique 30° hysteroscope (Karl Storz GmbH &Co KG, Tuttlingen, Germany; sheath diameter: 5mm) for diagnosis was done during the “implantation window” (5–7 days after ultrasound-confirmed ovulation) by experienced senior physicians and the uterine cavity examined thoroughly. Macroscopic signs of CE included the manifestation of diffuse or focal endometrial hyperemia, micropolyps (diameter <1 mm) and stromal edema (34)(**Figure 1**). Thickness of the endometrium was measured using transvaginal Doppler ultrasound. On the hysteroscopy day, blood samples were obtained for analysis of serum progesterone (P_4_) and estradiol (E_2_) levels. Instantly after hysteroscopy, all women were subjected to endometrial biopsy by inserting the tip of Pipelle sampler (Prodimed) into uterine fundus and moving it back and forth 4 times, each with a 90 degree rotation. Endometrial samples were rinsed thrice using sterile phosphate buffered-saline (PBS) and each sample separated into 2 aliquots, one for histological and immunohistochemistry assaying, another for our Biospecimen Bank which was stored at -80°C until use.

### Histology and Immunohistochemistry for CE diagnosis

Endometrial samples were fixed in neutral formaldehyde solution and later embedded in paraffin for histological analysis. The micro-sections were stained with hematoxylin and eosin. The histological examinations were performed by the single operator who was unaware of the hysteroscopic findings. Histological diagnosis of CE was following: superficial stromal edema, increased stromal density, pleomorphic stromal inflammatory infiltrate

dominated by lymphocytes, and plasma cells. Sections (5 μm) were cut and incubated with a 1:250 dilution of mouse anti-CD138 antibody (Gene Tech, clone B-A38 Shanghai, China).

We included only women with signs of CE at hysteroscopy whose diagnosis was confirmed by histology and immunohistochemistry for CD138.

CE diagnosis was based on microscopic observation of ≥1 CD138+ plasma cell/10 randomly selected high power fields (HPFs) in the stromal layer described before (1). However, the clinical significance of CD138+ 1–4/10 HPFs is controversial. A recent study found no significant difference in pregnancy outcomes in the CD138+ 1–4/10 HPFs group versus the control group (35). Another study found that the clinical pregnancy rate in patients with CD138+ ≥5/10 HPFs was markedly lower relative to patients with CD138+ 0–4/10 HPFs (64.6% vs. 44.74%; *P*=0.01) (5). However, no studies have examined the mechanisms underlying the effects of CE on pregnancy outcomes in patients with various levels of CD138+ plasma cells. Here, CE patients were classified into Group 1 (CD138+ 1–4/10 HPFs) and Group 2 (CD138+ ≥5/10 HPFs).

### Real time quantitative PCR

Isolation of total RNA from endometrial tissues was performed by a Trizol reagent (TaKaRa, Dalian, China). NanoDrop 2000 (Thermo Scientific-USA) was used to assess RNA purity. The HiScript III RT SuperMix (Vazyme Biotech Co., Ltd, Nangjing, China) was used for cDNA synthesis. ChamQ universal SYBR qPCR master mix (Vazyme Biotech Co.,Ltd, Nangjing, China) was used for real time quantitative PCR. The primers used in this study were: HIF1α: F-5’-atctcatccaagaagccctaac-3’, R-5’-gatcgtctggctgctgtaata-3’; VEGFA: F-5’-atcgagtacatcttcaagccat-3’, R-5’-gtgaggtttgatccgcataatc-3’; VEGFR2: F-5’-ggagcttaagaatgcatccttg-3’, R-5’-gatgctttccccaatacttgtc-3’; GAPDH (F-5’-ccgcctggagaaacctgccaag-3’, R-5’- caccaccctgttgctgtagccg-3’) (Sangon Biotech Co. Ltd., Shanghai, China). Relative gene expressions were determined by the 2^-ΔΔCt^ approach with GAPDH as reference gene. Assays were performed in triplicates.

### Western blot analysis

Endometrial tissues were lysed using RIPA buffer (Sparkjade, Jinan, China) and protein concentration evaluated by a BCA assay kit (Thermo Fisher Scientific). Equal concentrations of proteins were then separated on 10% SDS-PAGE gels, transferred onto polyvinylidene difluoride fluoride (PVDF) membrane (Sparkjade, Jinan, China) that were then blocked for 1 h using 5% skimmed milk in Tris-buffered saline with 0.05% at room temperature (RT). Thereafter, overnight incubation of membranes was done with rabbit primary antibodies against HIF1α, VEGFA, and VEGFR2 (all from Sangon Biotech, Shanghai, China) at 4°C. They were then incubated for 2 h with HRP-conjugated secondary antibodies at RT and signal developed by an ECL kit (Thermo Fisher Scientific). GAPDH was used as loading control. Image J (NIH, MD, USA) was used to measure band intensities.

### Immunohistochemistry staining

Immunohistochemical staining was done on 4-μm thick endometrial tissue sections fixed in formaldehyde and paraffin−embedded as previously reported (28). Briefly, xylene was used to deparaffinize the tissue sections while graded ethanol series was used to rehydrate them. Endogenous peroxidase activities were blocked using hydrogen peroxide (3%). To block non-specific binding, sections were incubated in 5% bovine serum albumin at RT. Next, incubation of the sections was done overnight with primary polyclonal rabbit antibodies against HIF1α, VEGFA, and VEGFR2 (all from Sangon Biotech, Shanghai, China) at 4°C. Then, they were incubated with conjugated anti-rabbit IgG secondary antibodies (Sangon Biotech, Shanghai, China) for 1 h at RT. Sections were then counterstained with Harris’ hematoxylin. For negative staining control samples, the primary antibodies were excluded. Sections were assessed by light microscopy (Leica) at a ×20 magnification. CD34 immunohistochemistry analysis using an anti-CD34 monoclonal antibody (Sangon Biotech, Shanghai, China, 1:100) was used to detect endothelial cells.

### Histochemical score

The levels of HIF1α, VEGFA, and VEGFR2 in endometrial sections was graded based on *H*-score as: *H* score =∑P_i_ (*i*+1). Whereby *i* is staining intensity (1, 2 and 3 respectively denote weak, moderate and strong) while P_i_ is the % of stained cells at every intensity (0%–100%). *H* scores were separately determined in stroma, luminal epithelium and glandular epithelium. Sections were independently scored by two examiners and any discrepancies between them, resolved through reexamination and consensus. Final HIF1α, VEGFA, and VEGFR2 staining scores were obtained from the mean score of three tests on each endometrial compartment (glandular epithelium, stroma, and luminal epithelium).

### Microvessel counting

CD34 staining was independently assessed under a light microscope (Leica; magnification: ×200, 0.754 mm^2^ per field) by three independent examiners. The 1^st^ field to be imaged was selected randomly while guaranteeing that it had a luminal epithelial border. Successive fields were acquired by moving one field to the right or left of original fields, keeping luminal epithelial borders in view to standardize endometrial area depth for counting micro-vessels. This process was repeated to capture 5 fields. Every brown-stained cell cluster or cell that had been clearly detached from neighboring microvessels and other connective tissue elements was taken as one countable vessel. Endometrial stroma area was measured in the same field in which blood vessel counts was determined and blood vessel counts presented per mm^2^ of the stroma. An observer-related mean was determined for every histologic section with the mean of the three observer-related means reported as final MVD for every sample.

### Statistical analysis

Statistical analyses were done on GraphPad Prism 8. Comparisons of normally distributed continuous variables was done by one-way ANOVA followed by Tukey’s *post hoc* test. The non-parametric Kruskal–Wallis test with Dunn’s multiple comparisons test was used to compare non-normally distributed data. Categorical variables were compared using Kruskal–Wallis χ^2^ test. Effects of antibiotics on HIF1α, VEGFA, and VEGFR2 expressions before and after treatment were compared using paired Student *t* test. *p*<0.05 indicated significant differences.

## Results

### Demographics

The participants demographic features are summarized on **Table 1**. The study group exhibited relatively high homogeneity. Age, body mass index (BMI), and menstrual cycle length did not differ markedly across all groups. Levels of E_2_ and P_4_, and endometrial thickness at the midluteal phase did not differ across the three groups.

**Table 1:** Demographic characteristics in women with CE or fertile controls.

	Control group (n=15)	Group 1 (n=53)	Group 2 (n=24)	*P* value
Age (y)	34.91 ± 1.67	36.51 ± 2.59	35.83 ± 2.31	0.291
BMI (Kg/m^2^)	22.32 ± 2.32	22.42 ± 1.54	21.92 ± 1.99	0.574
Menstrual cycle length (d)	28.10 ± 1.44	29.30 ± 1.52	30.20 ± 2.02	0.883
Endometrial thickness (mm)	8.93 ± 1.12	8.99 ± 1.54	8.87 ± 0.99	0.991
E_2_ (pg/mL)	209.66 ± 19.54	198.32 ± 16.97	188.11 ± 22.32	0.337
P_4_ (ng/mL)	8.77 ± 1.02	9.38 ± 2.03	8.99 ± 0.98	0.363
Infertility				
Primarily (n/%)	0	29	13	0.871^a^
Secondarily (n/%)	0	24	11	

Data are expressed as mean ± SD or n.

a Differences between groups were compared by χ^2^ test.

BMI, body mass index; E_2_, Estradiol; P_4_, Progesterone.

### The mRNA and protein expressions of HIF−1α, VEGFA, and VEGFR2 during implantation

**Figure 2:**
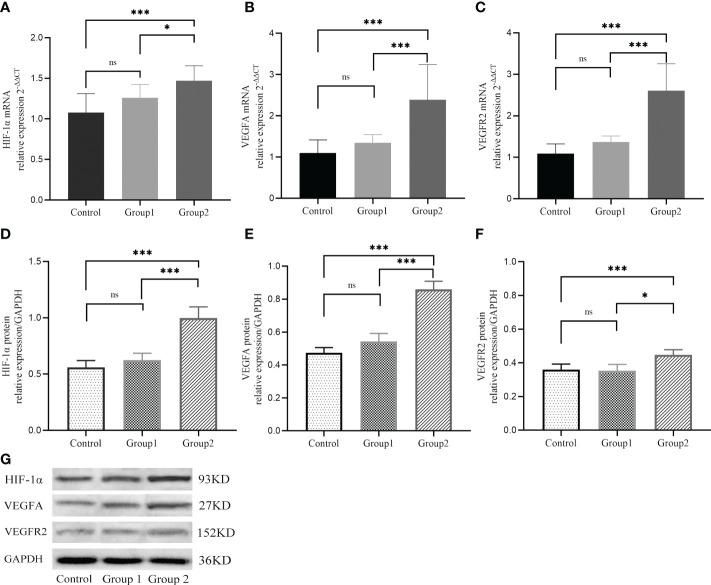
The mRNA **(A-C)** and protein **(D-G)** expressions of HIF−1α, VEGFA, and VEGFR2 during implantation in three groups. *, P < 0.05; ***, P < 0.01; ns, P > 0.05.

Compared with the control group and Group 1, the mRNA levels of HIF1α, VEGFA, and VEGFR2 were markedly high in Group 2 (*P*<0.05) but did not differ significantly between Group 1 and control group (*P*>0.05, **Figures 2A–C**). Similarly, western blot analysis showed that relative to control group and group 1, protein levels of HIF1α, VEGFA, and VEGFR2 were markedly higher in Group 2 (*P*<0.05), but did not differ between group 1 and control group (*P*>0.05, **Figures 2D–G**).

### Spatial levels of HIF1α, VEGFA, and VEGFR2 in peri-implantation endometrium

**Figure 3:**
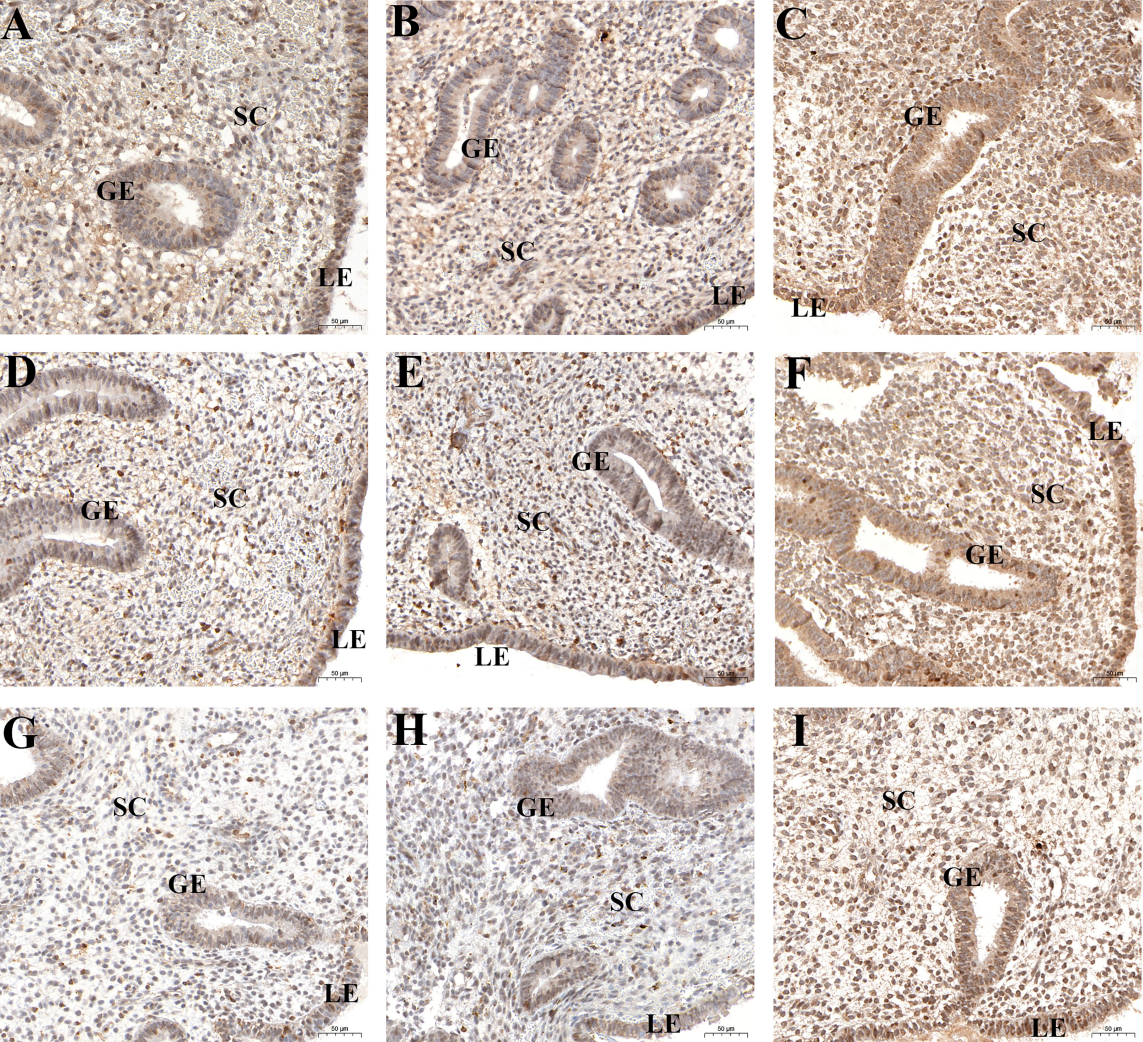
Spatial levels of HIF1α **(A-C)**, VEGFA **(D-F)**, and VEGFR2 **(G-I)** in peri-implantation endometrium from the fertile controls **(A, D, G)**, CD138+ 1–4/HPF group **(B, E, H)**, and CD138+ ≥5/HPF group **(C, F, I)**. Original magnification, ×200. Scale bar = 50μm. GE: glandular epithelium; LE: luminal epithelium; SC: stromal cell.

Immunohistochemical analysis revealed positive HIF1α, VEGFA, and VEGFR2 staining in all endometrial compartments in women with CE, as well as the fertile controls. HIF1α, VEGFA, and VEGFR2 staining was most intense in glandular epithelium, with the three groups exhibiting similar staining patterns (**Figure 3**). *H*-score analysis revealed that when compared with the CD138^-^ group and CD138+ 1–4/HPF group, the intensities of HIF1α, VEGFA, and VEGFR2 staining in luminal epithelium, glandular epithelium, and stroma were markedly higher in women with CD138+ ≥5/HPF (*P*<0.05). However, the *H*-scores for HIF1α, VEGFA, and VEGFR2 staining did not differ markedly between CD138^-^ and CD138+ 1–4/HPF groups (**Table 2**).

**Table 2:** The *H*-scores for HIF-1α, VEGFA and VEGFR2 in peri-implantation endometrium from women with CE or fertile controls.

				*P* value		
	Control group (n=15)	Group 1 (n=53)	Group 2 (n=24)	Control*Vs* Group 1	Group 1*Vs*Group 2	Control*Vs*Group 2
**Luminal epithelium**						
HIF-1α	41 (29-122)	48 (33-129)	107 (61-278)	0.521	0.012	0.005
VEGFA	55 (36-131)	53 (37-136)	111 (55-262)	0.613	0.024	0.011
VEGFR2	24 (15-82)	22 (29-92)	94 (18-190)	0.732	0.197	0.037
**Glandular epithelium**						
HIF-1α	167 (103-302)	173 (99-306)	247 (114-339)	0.651	0.032	0.028
VEGFA	162 (107-305)	179 (104-302)	263 (109-344)	0.729	0.044	0.036
VEGFR2	101 (59-207)	108 (86-218)	214 (93-302)	0.596	0.038	0.014
**Stroma**						
HIF-1α	36 (22-99)	38 (19-110)	72 (18-130)	0.577	0.049	0.042
VEGFA	43 (29-114)	49 (31-126)	83 (20-138)	0.693	0.044	0.022
VEGFR2	19 (17-97)	21 (18-92)	84 (58-164)	0.805	0.031	0.012

Data are expressed as median (range). Groups were compared by non-parametric Kruskal-Wallis test with Dunn’s multiple comparisons test.

### Microvascular density (MVD)

**Figure 4:**
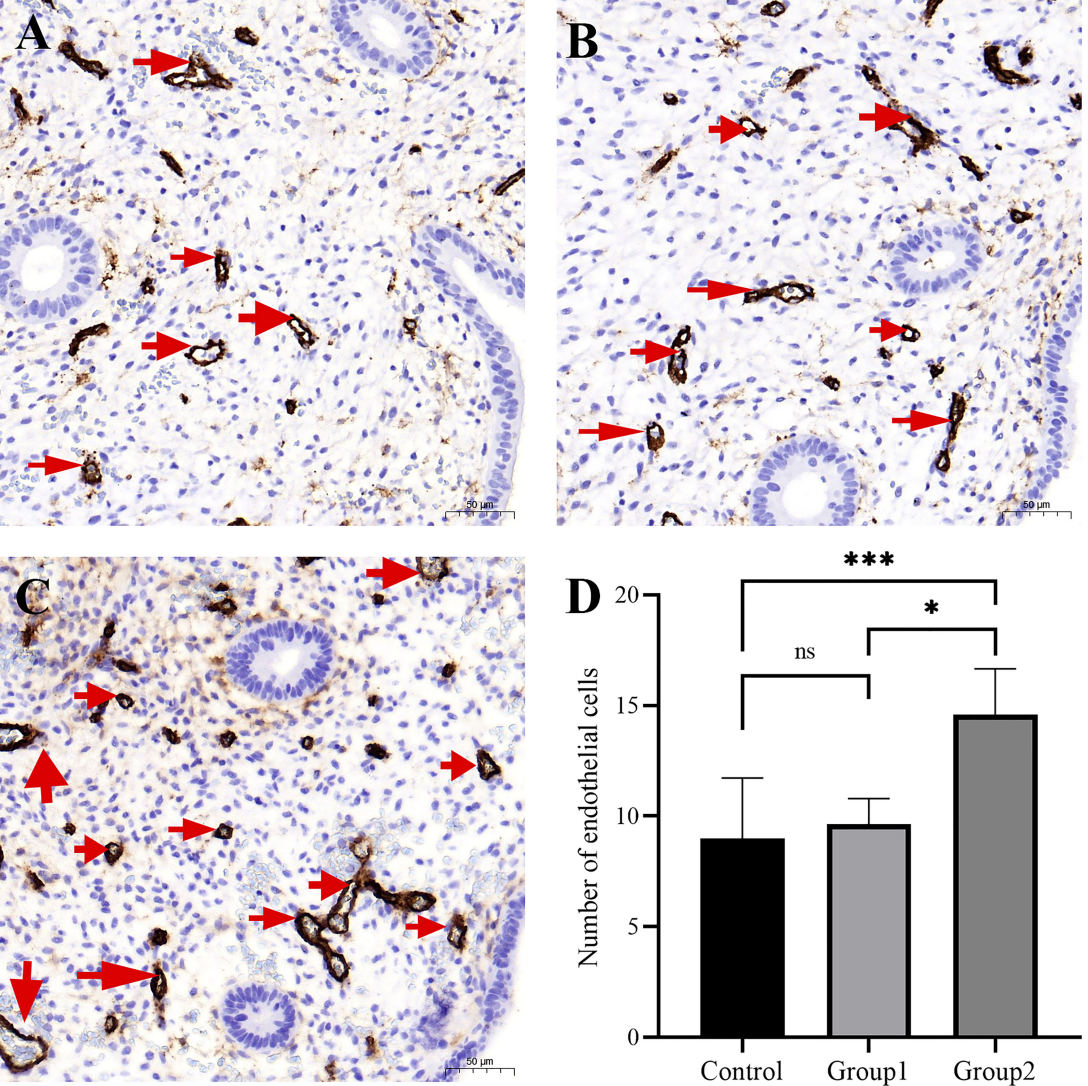
The micro-blood vessels in endometrial biopsies were determined by CD34–stained sections. Representative images of blood vessels localized in endometrium (arrows) from the fertile controls **(A)**, CD138+ 1–4/10 HPFs group **(B)**, and CD138+ ≥5/10 HPFs group **(C)** are shown. Original magnification, ×200. Scale bar = 50μm. The CD138+ ≥5/10 HPFs group exhibited the highest MVD values **(D)**. *, P < 0.05; ***, P < 0.01; ns, P > 0.05.

Brown-staining for CD34 indicated endothelial cells or endothelial cell clusters in the endometrial stromal layer, which was considered to be a single, countable vessel. Image J analysis showed that the density of CD34-positive cells in Group 2 was higher than in control group and Group 1. However, the density of CD34-positive cells did not markedly differ between Group 1 and control group (*P*>0.05, **Figures 4A-C**). The CD138+ ≥5/HPF group exhibited the highest MVD values (**Figure 4D**), indicating that inflammation level influences endometrial angiogenesis.

### Changes of HIF−1α, VEGFA and VEGFR2 in CE patients after antibiotics therapy

**Figure 5:**
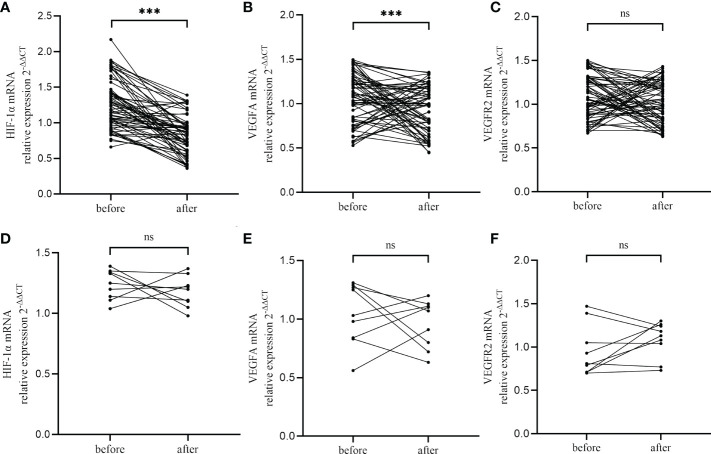
Changes of HIF−1α **(A, D)**, VEGFA **(B, E)** and VEGFR2 **(C, F)** in CE patients after antibiotics therapy, including CE-cured patients **(A-C)** and CE-persistent patients **(D-F)**. ***, P < 0.01; ns, P > 0.05.

CE patients were orally treated with the first-line antibiotics, metronidazole (400 mg, once daily for 14 days) and doxycycline (100 mg, twice daily). Following the first course of antibiotics, 65 of 74 participants (87.8%) who underwent a second endometrial biopsy (in the next “implantation window” phase) were found to be recovered, as revealed by the absence of stromal plasma cells. The nine (12.2%) patients who still presented with CD138+/10 HPFs were categorized as having persistent CE. RT-qPCR analyzed the impact of antibiotics on the expression of HIF1α, VEGFA, and VEGFR2 in CE patients, and showed that levels of HIF1α and VEGFA were markedly reduced in patients who recovered from CE when compared with paired patients before treatment (*P<*0.001 and *P*=0.011, respectively, **Figures 5A, B**). Nevertheless, levels of HIF1α and VEGFA did not differ markedly between patients with persistent CE (*P*=0.41 and *P*=0.49, respectively, **Figure 5D, E**). VEGFR2 levels did not differ considerably prior to and after antibiotic treatment (*P*=0.16 and *P*=0.21, respectively, **Figures 5C, F**).

## Discussion

Although CE is well known to adversely affect endometrial receptivity, the underlying mechanisms are poorly understood. Appropriate angiogenesis is considered to be an important marker of endometrial receptivity. Furthermore, HIF1α, an indicator of oxygen homeostasis, regulates the endometrial microenvironment and drives the transcription of angiogenic and metabolic genes. This is the first study to explore the effects of CE on HIF1α expression and endometrial vascularity.

Here, we find that endometrial HIF1α mRNA and protein levels were markedly elevated in infertile CE patients with CD138 ≥5/10 HPFs, but did not differ significantly between normal fertile women without CE and CE patients with CD138 1–4/10 HPFs. This observation is consistent with findings from other CE-associated disorders like endometriosis (36) and recurrent miscarriage. In endometriosis, Zhang et al. reported significantly higher HIF1α levels in the eutopic endometrium when compared with the normal endometrium (37). Another study observed in women with recurrent miscarriage, endometrial HIF1α protein levels increased significantly around the time of embryo implantation (38). The endometrium has complex oxygen requirements that change after implantation. Prior to implantation, dissolved oxygen concentrations are relatively low on endometrium surfaces, relative to inner portions of the endometrium and myometrium (23). Oxygen concentration diminishes with distance from blood vessels (39). Furthermore, before implantation, development of the embryo occurs in hypoxic environments (25). Thus, hypoxic environments of pre-implantation endometrium are appropriate for human embryogenesis. However, after implantation and angiogenesis, endometrial oxygen levels rises significantly (40), leading to an aerobic environment with adequate oxygen levels for embryonic development. Thus, the initial hypoxic endometrial microenvironment changes over time, which results in dysregulated physiological functions. Based on our findings and previous reports, we hypothesize that in CE patients, endometrial oxygen levels remain low after embryonic implantation, resulting in a hypoxic microenvironment without sufficient oxygen for normal embryonic development, and which negatively affects endometrial receptivity for the embryo. However, the exact mechanisms by which CE contributes to a persistently hypoxic microenvironment requires further investigation.

Our findings also revealed that relative to women without CE and CE patients with CD138 1–4/10 HPFs, CE patients with CD138 ≥5/10 HPFs had significantly higher levels of VEGFA and its receptor, VEGFR2. Consistent with our findings, Cicinelli, E. et al. found that when compared with patients without CE, VEGFA mRNA levels were markedly elevated in proliferative endometria of CE patients. However, in contrast with our study, using high-throughput RT PCR, Cicinelli, E. et al. did not establish marked differences in endometrial VEGFA mRNA levels during the implantation window in CE patients versus controls. The discrepancy may be because of differences in CE definition based on fluid hysteroscopy and histology, small sample size, and ethnic differences in the study by Cicinelli, E. et al. Our study, involving a relatively large sample size and various experimental techniques, found that VEGFA and its main receptor, VEGFR2, were markedly upregulated in CE patients, implying that CE may be associated with excessive endometrial angiogenesis. Moreover, IHC analysis confirmed that CE patients have superfluous endometrial vascular density (CD34-positive cells), which in turn, impairs endometrial receptivity. Based on ERA testing, a recent study reported that the individual WOI of 62.5% (10/16 cases) CE patients might be brought forward by the CE (22). Taken together, these findings indicate that the upregulation of VEGFA and VEGFR2, and increased endometrial MVD in CE patients may cause embryo–endometrial asynchrony.

We noted a significant increase in levels of endometrial HIF1α, VEGFA, and VEGFR2 in all compartments, including the luminal epithelium, glandular epithelium, and endometrial stroma in women with CD138 ≥5/10 HPFs. Elevated levels of plasma cells in endometrial tissue are correlated with a marked increase in expressions of master regulators of oxygen homeostasis and vasculogenesis, HIF−1α, VEGFA and VEGFR2. It is known that successful implantation, placentation, and gestation need synchronized vascular development and adaptation in every endometrial compartment. Thus, we postulated that persistent chronic inflammation may cause endometrial luminal, stromal cells, and glands to undergo holistic changes that disrupt normal endometrial physiology, thereby impairing reproductive capacity in CE patients. Additionally, CE may often be asymptomatic, or it may be accompanied by irregular vaginal bleeding and/or increased leukorrhea. Moreover, endometrial hyperemia, the presence of diffuse micropolyps (diameter <1mm), or polypoid endometria are frequently observed in CE patients during hysteroscopy. Therefore, the prevalence of known spatial HIF1α, VEGFA, and VEGFR2 overexpression may explain the impaired endometrial receptivity associated with CE and support the hypothesis that CE is pathologically involved in the development of various endometrial disorders, including abnormal bleeding and proliferative diseases like hyperemia, micropolyps, and hyperplastic lesions.

Currently, standardized guidelines for CE diagnosis are lacking. Particularly, consensus on the minimum number of stromal plasma cells indicative of clinical CE is lacking. Different reproductive medical centers use different criteria to determine the density of plasmacytes. Some studies have applied the stringent criterion of >5 CD138+ cells in at least 1 of 3 section levels in endometrial samples (5, 17). However, most studies apply ≥1 CD138+ cells in 1 microscopic HPF as the diagnostic criterion (9, 36, 41, 42). Studies have not determined if variations in CD138+ cells/HPF are responsible for the different pathomechanisms in CE patients. We established that endothelial MVD and expression patterns of HIF1α, VEGFA, and VEGFR2 do not differ between women without CE and CE patients with CD138 1–4/10 HPFs, but we did find significant elevations in CE patients with CD138+ ≥5/10 HPFs. These findings are consistent with previous findings that pregnancy outcomes are not affected in women with CD138+ 1–4/10 HPFs (35). Based on the finding above, we postulate that different numbers of endometrial CD138+ cells may have different pathological mechanisms. This possibility warrants further investigation.

Notably, we found that a single antibiotic treatment course significantly reduced the expression of HIF1α and VEGFA in cured CE patients. However, for patients with persistent CE, the expression levels of HIF1α and VEGFA did not differ significantly after antibiotic treatment when compared to before treatment. Additionally, antibiotics did not appear to affect VEGFR2 expression, whether or not CE was cured. These observations indicate that first line antibiotics (doxycycline plus metronidazole) mainly influence HIF−1α and VEGFA but not VEGFR2. Most studies indicate that antibiotics are effective against CE and therefore improve reproductive outcomes (43). Thus, the recovery of endometrial HIF1α and VEGFA expression levels in cured CE patients may result from successful treatment and may improve pregnancy outcomes.

This study has limitations. Because this is a retrospective, single-center study, clinical and demographic data of the study participants were incomplete. Therefore, some factors that may confound our conclusions may have been missed. Thus, prospective multicenter clinical studies are needed to validate our observations. Additionally, because this is the first study in this field, we only used representative factors from the hypoxia-inducible factor family and the vascular endothelial growth factor family to study the status of hypoxia and vascularization. Hence, other important factors may be involved. Finally, the participants’ pregnancy outcomes (e.g., implantation rate and pregnancy rate) were not assessed after antibiotic treatment. Thus, we did not establish the clinical prognostic value of treatment with antibiotics.

## Conclusion

Here, we report that endometrial upregulation of HIF1α, VEGFA, and VEGFR2, as well as excessive vascularization in the peri-implantation endometrium, may reduce endometrial receptivity in infertile CE patients. Notably, although endometrial hypoxic and angiogenic patterns in patients with CD138+ 1–4/HPF were similar to those of participants without CE, they were markedly aberrant in women with CD138+ ≥5/HPF. Finally, we report that antibiotics-mediated remodeling of hypoxic and angiogenic homeostasis in the endometrium may improve reproductive outcomes. Our findings offer new insights into reduced endometrial receptivity in CE-associated infertility.

## Data availability statement

The raw data supporting the conclusions of this article will be made available by the authors, without undue reservation.

## Ethics statement

The studies involving human participants were reviewed and approved by the ethical committee of Yantai Yuhuangding Hospital Affiliated to Qingdao University. The patients/participants provided their written informed consent to participate in this study.

## Author contributions

ZL, XL, and FL conceived and designed the study. YS and LY performed the real time quantitative PCR and the western blot. PZ and WZ contributed to the statistical analysis. DM completed immunohistochemistry staining. XW and SL analyzed histochemical score, and DM, XW and SL completed microvessel counting. ZL, XL, and HB wrote the manuscript. HB was responsible for the revision of articles. All authors reviewed the manuscript.

## Funding

The study was funded by the Key Technology Research and Development Program of Shandong, China (No. 2019JZZY020902).

## Acknowledgments

We thank all the subjects for taking part in this study. The authors also extend our thanks to all staff involved at the clinics.

## Conflict of interest

The authors declare that the research was conducted in the absence of any commercial or financial relationships that could be construed as a potential conflict of interest.

## Publisher’s note

All claims expressed in this article are solely those of the authors and do not necessarily represent those of their affiliated organizations, or those of the publisher, the editors and the reviewers. Any product that may be evaluated in this article, or claim that may be made by its manufacturer, is not guaranteed or endorsed by the publisher.

## References

[1] Cicinelli E Resta L Loizzi V Pinto V Santarsiero C Cicinelli R. Antibiotic therapy versus no treatment for chronic endometritis: A case-control study. Fertil Steril (2021) 115(6):1541–8. doi: 10.1016/j.fertnstert.2021.01.018 33722376

[2] Drizi A Djokovic D Lagana AS van Herendael B. Impaired inflammatory state of the endometrium: A multifaceted approach to endometrial inflammation. current insights and future directions. Prz Menopauzalny (2020) 19(2):90–100. doi: 10.5114/pm.2020.97863 32802019 PMC7422289

[3] Crum CP Egawa K Fenoglio CM Richart RM. Chronic endometritis: the role of immunohistochemistry in the detection of plasma cells. Am J Obstet Gynecol (1983) 147(7):812–5. doi: 10.1016/0002-9378(83)90045-5 6359886

[4] Moreno I Cicinelli E Garcia-Grau I Gonzalez-Monfort M Bau D Vilella F. The diagnosis of chronic endometritis in infertile asymptomatic women: a comparative study of histology, microbial cultures, hysteroscopy, and molecular microbiology. Am J Obstet Gynecol (2018) 218(6):602.e601–2.e616. doi: 10.1016/j.ajog.2018.02.012 29477653

[5] Li Y Xu S Yu S Huang C Lin S Chen W. Diagnosis of chronic endometritis: How many CD138(+) cells/HPF in endometrial stroma affect pregnancy outcome of infertile women? Am J Reprod Immunol (2021) 85(5):e13369. doi: 10.1111/aji.13369 33152123

[6] Johnston-MacAnanny EB Hartnett J Engmann LL Nulsen JC Sanders MM Benadiva CA. Chronic endometritis is a frequent finding in women with recurrent implantation failure after *in vitro* fertilization. Fertil Steril (2010) 93(2):437–41. doi: 10.1016/j.fertnstert.2008.12.131 19217098

[7] Kitaya K Tada Y Hayashi T Taguchi S Funabiki M Nakamura Y. Comprehensive endometrial immunoglobulin subclass analysis in infertile women suffering from repeated implantation failure with or without chronic endometritis. Am J Reprod Immunol (2014) 72(4):386–91. doi: 10.1111/aji.12277 24898900

[8] Liu Y Chen X Huang J Wang CC Yu MY Laird S. Comparison of the prevalence of chronic endometritis as determined by means of different diagnostic methods in women with and without reproductive failure. Fertil Steril (2018) 109(5):832–9. doi: 10.1016/j.fertnstert.2018.01.022 29778382

[9] McQueen DB Bernardi LA Stephenson MD. Chronic endometritis in women with recurrent early pregnancy loss and/or fetal demise. Fertil Steril (2014) 101(4):1026–30. doi: 10.1016/j.fertnstert.2013.12.031 24462055

[10] McQueen DB Perfetto CO Hazard FK Lathi RB. Pregnancy outcomes in women with chronic endometritis and recurrent pregnancy loss. Fertil Steril (2015) 104(4):927–31. doi: 10.1016/j.fertnstert.2015.06.044 26207958

[11] Bouet PE El Hachem H Monceau E Gariepy G Kadoch IJ Sylvestre C. Chronic endometritis in women with recurrent pregnancy loss and recurrent implantation failure: Prevalence and role of office hysteroscopy and immunohistochemistry in diagnosis. Fertil Steril (2016) 105(1):106–10. doi: 10.1016/j.fertnstert.2015.09.025 26456229

[12] Morimune A Kimura F Nakamura A Kitazawa J Takashima A Amano T. The effects of chronic endometritis on the pregnancy outcomes. Am J Reprod Immunol (2021) 85(3):e13357. doi: 10.1111/aji.13357 33020952

[13] Cicinelli E Matteo M Tinelli R Lepera A Alfonso R Indraccolo U. Prevalence of chronic endometritis in repeated unexplained implantation failure and the IVF success rate after antibiotic therapy. Hum Reprod (2015) 30(2):323–30. doi: 10.1093/humrep/deu292 25385744

[14] Kitaya K Matsubayashi H Takaya Y Nishiyama R Yamaguchi K Takeuchi T. Live birth rate following oral antibiotic treatment for chronic endometritis in infertile women with repeated implantation failure. Am J Reprod Immunol (2017) 78(5). doi: 10.1111/aji.12719 28608596

[15] Song D Feng X Zhang Q Xia E Xiao Y Xie W. Prevalence and confounders of chronic endometritis in premenopausal women with abnormal bleeding or reproductive failure. Reprod BioMed Online (2018) 36(1):78–83. doi: 10.1016/j.rbmo.2017.09.008 29111313

[16] Kitaya K Yasuo T. Aberrant expression of selectin e, CXCL1, and CXCL13 in chronic endometritis. Mod Pathol (2010) 23(8):1136–46. doi: 10.1038/modpathol.2010.98 20495539

[17] Li Y Yu S Huang C Lian R Chen C Liu S. Evaluation of peripheral and uterine immune status of chronic endometritis in patients with recurrent reproductive failure. Fertil Steril (2020) 113(1):187–96.e181. doi: 10.1016/j.fertnstert.2019.09.001 31718829

[18] Di Pietro C Cicinelli E Guglielmino MR Ragusa M Farina M Palumbo MA. Altered transcriptional regulation of cytokines, growth factors, and apoptotic proteins in the endometrium of infertile women with chronic endometritis. Am J Reprod Immunol (2013) 69(5):509–17. doi: 10.1111/aji.12076 23351011

[19] Cicinelli E Vitagliano A Loizzi V De Ziegler D Fanelli M Bettocchi S. Altered gene expression encoding cytochines, grow factors and cell cycle regulators in the endometrium of women with chronic endometritis. Diagnostics (Basel) (2021) 11(3):471. doi: 10.3390/diagnostics11030471 33800186 PMC7999985

[20] Wu D Kimura F Zheng L Ishida M Niwa Y Hirata K. Chronic endometritis modifies decidualization in human endometrial stromal cells. Reprod Biol Endocrinol (2017) 15(1):16. doi: 10.1186/s12958-017-0233-x 28259137 PMC5336610

[21] Wang WJ Zhang H Chen ZQ Zhang W Liu XM Fang JY. Endometrial TGF-beta, IL-10, IL-17 and autophagy are dysregulated in women with recurrent implantation failure with chronic endometritis. Reprod Biol Endocrinol (2019) 17(1):2. doi: 10.1186/s12958-018-0444-9 30606202 PMC6317248

[22] Kuroda K Horikawa T Moriyama A Nakao K Juen H Takamizawa S. Impact of chronic endometritis on endometrial receptivity analysis results and pregnancy outcomes. Immun Inflammation Dis (2020) 8(4):650–8. doi: 10.1002/iid3.354 PMC7654412 32969185

[23] Chen X Man GCW Liu Y Wu F Huang J Li TC. Physiological and pathological angiogenesis in endometrium at the time of embryo implantation. Am J Reprod Immunol (2017) 78(2). doi: 10.1111/aji.12693 28466568

[24] Kingdom JC Kaufmann P. Oxygen and placental vascular development. Adv Exp Med Biol (1999) 474:259–75. doi: 10.1007/978-1-4615-4711-2_20 10635006

[25] Red-Horse K Zhou Y Genbacev O Prakobphol A Foulk R McMaster M. Trophoblast differentiation during embryo implantation and formation of the maternal-fetal interface. J Clin Invest (2004) 114(6):744–54. doi: 10.1172/JCI22991 PMC516273 15372095

[26] Critchley HO Osei J Henderson TA Boswell L Sales KJ Jabbour HN. Hypoxia-inducible factor-1alpha expression in human endometrium and its regulation by prostaglandin e-series prostanoid receptor 2 (EP2). Endocrinology (2006) 147(2):744–53. doi: 10.1210/en.2005-1153 16282352

[27] Semenza GL. Expression of hypoxia-inducible factor 1: mechanisms and consequences. Biochem Pharmacol (2000) 59(1):47–53. doi: 10.1016/s0006-2952(99)00292-0 10605934

[28] Zhao D Qu D Dai H Liu Y Jiang L Huang X. Effects of hypoxiainducible factor1alpha on endometrial receptivity of women with polycystic ovary syndrome. Mol Med Rep (2018) 17(1):41–21. doi: 10.3892/mmr.2017.7890 29115598

[29] Guo X Yi H Li TC Wang Y Wang H Chen X. Role of vascular endothelial growth factor (VEGF) in human embryo implantation: Clinical implications. Biomolecules (2021) 11(2):253. doi: 10.3390/biom11020253 33578823 PMC7916576

[30] Walter LM Rogers PA Girling JE. Differential expression of vascular endothelial growth factor-a isoforms in the mouse uterus during early pregnancy. Reprod BioMed Online (2010) 21(6):803–11. doi: 10.1016/j.rbmo.2010.07.005 21050818

[31] Rogers PA Martinez F Girling JE Lederman F Cann L Farrell E. Influence of different hormonal regimens on endometrial microvascular density and VEGF expression in women suffering from breakthrough bleeding. Hum Reprod (2005) 20(12):3341–7. doi: 10.1093/humrep/dei239 16085661

[32] Chen L Quan S Ou XH Kong L. Decreased endometrial vascularity in patients with antiphospholipid antibodies-associated recurrent miscarriage during midluteal phase. Fertil Steril (2012) 98(6):1495–502.e1491. doi: 10.1016/j.fertnstert.2012.08.006 22959454

[33] Chen X Jin X Liu L Man CW Huang J Wang CC. Differential expression of vascular endothelial growth factor angiogenic factors in different endometrial compartments in women who have an elevated progesterone level before oocyte retrieval, during *in vitro* fertilization-embryo transfer treatment. Fertil Steril (2015) 104(4):1030–6. doi: 10.1016/j.fertnstert.2015.06.021 26143364

[34] Cicinelli E Vitagliano A Kumar A Lasmar RB Bettocchi S Haimovich S. Unified diagnostic criteria for chronic endometritis at fluid hysteroscopy: proposal and reliability evaluation through an international randomized-controlled observer study. Fertil Steril (2019) 112(1):162–73.e162. doi: 10.1016/j.fertnstert.2019.03.004 31104760

[35] Xiong Y Chen Q Chen C Tan J Wang Z Gu F. Impact of oral antibiotic treatment for chronic endometritis on pregnancy outcomes in the following frozen-thawed embryo transfer cycles of infertile women: a cohort study of 640 embryo transfer cycles. Fertil Steril (2021) 116(2):413–21. doi: 10.1016/j.fertnstert.2021.03.036 33926717

[36] Cicinelli E Trojano G Mastromauro M Vimercati A Marinaccio M Mitola PC. Higher prevalence of chronic endometritis in women with endometriosis: a possible etiopathogenetic link. Fertil Steril (2017) 108(2):289–95.e281. doi: 10.1016/j.fertnstert.2017.05.016 28624114

[37] Zhang L Xiong W Li N Liu H He H Du Y. Estrogen stabilizes hypoxia-inducible factor 1alpha through G protein-coupled estrogen receptor 1 in eutopic endometrium of endometriosis. Fertil Steril (2017) 107(2):439–47. doi: 10.1016/j.fertnstert.2016.11.008 PMC5292292 27939762

[38] Chen X Jiang L Wang CC Huang J Li TC. Hypoxia inducible factor and microvessels in peri-implantation endometrium of women with recurrent miscarriage. Fertil Steril (2016) 105(6):1496–502.e1494. doi: 10.1016/j.fertnstert.2016.02.032 27018158

[39] Rodesch F Simon P Donner C Jauniaux E. Oxygen measurements in endometrial and trophoblastic tissues during early pregnancy. Obstet Gynecol (1992) 80(2):283–5. 1635745

[40] Matsumoto L Hirota Y Saito-Fujita T Takeda N Tanaka T Hiraoka T. HIF2alpha in the uterine stroma permits embryo invasion and luminal epithelium detachment. J Clin Invest (2018) 128(7):3186–97. doi: 10.1172/JCI98931 PMC6025968 29911998

[41] Cicinelli E Matteo M Tinelli R Pinto V Marinaccio M Indraccolo U. Chronic endometritis due to common bacteria is prevalent in women with recurrent miscarriage as confirmed by improved pregnancy outcome after antibiotic treatment. Reprod Sci (2014) 21(5):640–7. doi: 10.1177/1933719113508817 PMC3984485 24177713

[42] Yang R Du X Wang Y Song X Yang Y Qiao J. The hysteroscopy and histological diagnosis and treatment value of chronic endometritis in recurrent implantation failure patients. Arch Gynecol Obstet (2014) 289(6):1363–9. doi: 10.1007/s00404-013-3131-2 24395012

[43] Vitagliano A Saccardi C Noventa M Di Spiezio Sardo A Saccone G Cicinelli E. Effects of chronic endometritis therapy on *in vitro* fertilization outcome in women with repeated implantation failure: a systematic review and meta-analysis. Fertil Steril (2018) 110(1):103–12.e101. doi: 10.1016/j.fertnstert.2018.03.017 29908776

